# *Staphylococcus aureus* Complex in the Straw-Colored Fruit Bat (*Eidolon helvum*) in Nigeria

**DOI:** 10.3389/fmicb.2018.00162

**Published:** 2018-02-13

**Authors:** Ayodele Olatimehin, Adebayo O. Shittu, Francis C. Onwugamba, Alexander Mellmann, Karsten Becker, Frieder Schaumburg

**Affiliations:** ^1^Department of Microbiology, Obafemi Awolowo University, Ile-Ife, Nigeria; ^2^Institute of Medical Microbiology, University Hospital Münster, Münster, Germany; ^3^Institute of Hygiene, University Hospital Münster, Münster, Germany

**Keywords:** *Staphylococcus aureus*, *Staphylococcus schweitzeri*, *Staphylococcus argenteus*, Africa, *Eidolon helvum*

## Abstract

Bats are economically important animals and serve as food sources in some African regions. They can be colonized with the *Staphylococcus aureus* complex, which includes *Staphylococcus schweitzeri* and *Staphylococcus argenteus*. Fecal carriage of *S. aureus* complex in the straw-colored fruit bat (*Eidolon helvum*) has been described. However, data on their transmission and adaptation in animals and humans are limited. The aim of this study was to investigate the population structure of the *S. aureus* complex in *E. helvum* and to assess the geographical spread of *S. aureus* complex among other animals and humans. Fecal samples were collected from *E. helvum* in Obafemi Awolowo University, Ile-Ife, Nigeria. The isolates were characterized by antimicrobial susceptibility testing, *spa* typing and multilocus sequence typing (MLST). Isolates were screened for the presence of *lukS*/*lukF*-PV and the immune evasion cluster (*scn, sak, chp*) which is frequently found in isolates adapted to the human host. A Neighbor-Joining tree was constructed using the concatenated sequences of the seven MLST genes. A total of 250 fecal samples were collected and 53 isolates were included in the final analysis. They were identified as *S. aureus* (*n* = 28), *S. schweitzeri* (*n* = 11) and *S. argenteus* (*n* = 14). Only one *S. aureus* was resistant to penicillin and another isolate was intermediately susceptible to tetracycline. The *scn, sak*, and *chp* gene were not detected. Species-specific MLST clonal complexes (CC) were detected for *S. aureus* (CC1725), *S. argenteus* (CC3960, CC3961), and *S. schweitzeri* (CC2463). STs of *S. schweitzeri* from this study were similar to STs from bats in Nigeria (ST2464) and Gabon (ST1700) or from monkey in Côte d'Ivoire (ST2058, ST2072). This suggests host adaptation of certain clones to wildlife mammals with a wide geographical spread in Africa. In conclusion, there is evidence of fecal carriage of members of *S. aureus* complex in *E. helvum*. *S. schweitzeri* from bats in Nigeria are closely related to those from bats and monkeys in West and Central Africa suggesting a cross-species transmission and wide geographical distribution. The low antimicrobial resistance rates and the absence of the immune evasion cluster suggests a limited exposure of these isolates to humans.

## Introduction

Bats are pollinators of economically important plants and a source of animal protein (Boyles et al., 2011). However, they are also reservoirs and vectors for zoonotic pathogens such as Ebola virus, Marburg virus, Nipah virus, Rabies virus, or coronavirus (Plowright et al., 2015; Allocati et al., 2016; Streicker and Allgeier, 2016). A key factor in the transmission of zoonotic pathogens is the overlap of the habitat of reservoirs and the recipient host. Drivers for transmission are therefore deforestation, intensified farming, livestock production, or the consumption of so-called bush meat (e.g., bats, antelopes, reptiles, rodents) (Wolfe et al., 2005; Streicker and Allgeier, 2016).

Although the investigation of bats as reservoirs for zoonotic pathogens is mainly focused on viruses, bacteria of medical importance such as *Bartonella* spp. (Cicuttin et al., 2017; Stuckey et al., 2017), *Leptospira* sp. (Dietrich et al., 2015), *Rickettsia* sp. (Cicuttin et al., 2017), or *Borrelia* sp. have also been described (Brook and Dobson, 2015). Two reports have noted the *Staphylococcus aureus* complex colonization of the nasopharynx or intestinal tract of fruit bats (Akobi et al., 2012; Held et al., 2017). Members of the *S. aureus* complex include *S. aureus, Staphylococcus argenteus*, and *Staphylococcus schweitzeri* (Tong et al., 2015). *S. argenteus* can cause several infections in humans such as skin and soft tissue infection or bacteremia (Jenney et al., 2014; Dupieux et al., 2015; Chantratita et al., 2016). In contrast, *S. schweitzeri* colonize mainly non-human primates and bats (Akobi et al., 2012; Schaumburg et al., 2015; Held et al., 2017). Colonization of *S. schweitzeri* in humans has been reported in three cases with a possible zoonotic source (Ateba Ngoa et al., 2012; Okuda et al., 2016). However, human infections with *S. schweitzeri* have not been reported yet.

A large population of the straw-colored fruit bat (*Eidolon helvum*) roost on trees of the main campus of Obafemi Awolowo University, Ile-Ife, Nigeria (Okon, 1974). They migrate seasonally and abandon their colonies during the rainy season. Information on intestinal colonization by members of the *S. aureus* complex in *E. helvum* and the level of transmission to humans are limited. The aim of this study was to analyze the population structure of *S. aureus* complex in *E. helvum* and to assess the spread of *S. aureus* complex among other animals and humans.

## Materials and methods

### Ethics

No ethical clearance was necessary as animals were not captured and no invasive samples were taken. The authors complied with all of the legal requirements pertaining to the locations in which the work was done.

### Fecal samples

**Figure 1:**
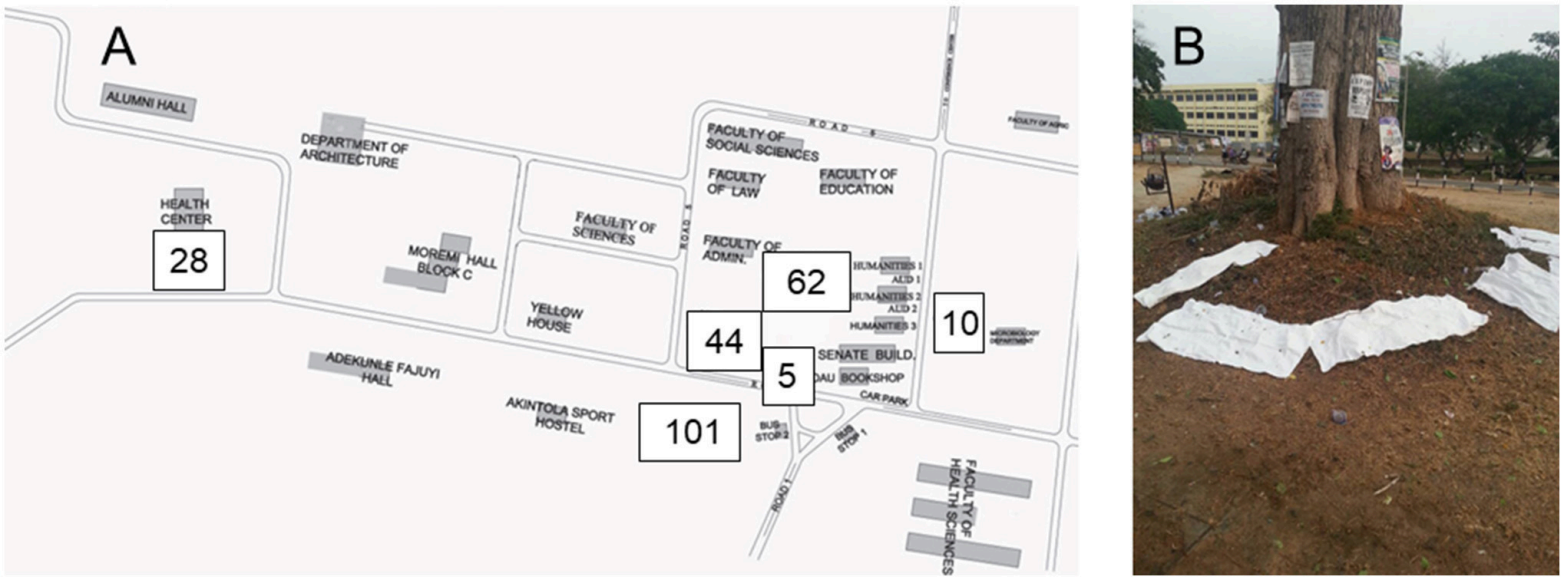
Collection of feacal samples. Samples were collected at six sites on the Obafemi Awolowo University campus in Ile-Ife, Nigeria **(A)**. The number of samples are indicated for each sampling site. For the collection of samples, sterile cotton materials (36 × 45 inches) were placed under roosting sites of Straw Colored Fruit Bats (*E. helvum*, **B)**. Swabs from feacal droppings were screened for *Staphylococcus aureus* complex.

Fecal samples from *E. helvum* were obtained (between 6 and 7 a.m.) from six different roost sites (Figure 1) and processed as previously described (Akobi et al., 2012). In brief, sterilized (washed with detergent, rinsed with water, sealed, autoclaved and dried in a hot air oven for 3 h) pieces of cotton material (36 × 45 inches) were spread under the roosting trees of *E. helvum*. Fecal samples were transferred from the cotton materials using sterile swab. The sampling period was from October 2015 to June 2016.

The samples were cultured in nutrient broth (Merck, Darmstadt, Germany) overnight at 37°C. Thereafter, a 10 μl of the broth was cultured on mannitol salt agar (37°C, 48 h).

### Bacterial isolates

*Staphylococcus aureus* complex isolates were presumptively identified based on Gram stain, a positive catalase, coagulase, and DNase reaction. Species confirmation was done in Germany using matrix-assisted laser desorption ionization time-of-flight mass spectrometry (MALDI-TOF MS; Bruker Daltonics, Bremen, Germany). A *S. aureus* specific thermostable nuclease (*nuc*) PCR was used to distinguish *S. aureus* from *S. schweitzeri*/*S. argenteus* (Brakstad et al., 1992; Schaumburg et al., 2014b).

Susceptibility was tested using Vitek2 automated system (bioMérieux, Marcy l'Etoile, France) and EUCAST clinical breakpoints (Version 7.1). Resistance to penicillin was confirmed by the detection of the *bla* gene (Kaase et al., 2008). Factors that mediate immune evasion of *S. aureus* in humans (*scn, sak, chp*) were tested by PCR to assess an adaptation of isolates from bats to the human host (van Wamel et al., 2006). All isolates were screened for *lukS*-PV/*lukF*-PV encoding the Panton-Valentine leukocidin (PVL), which is one of the most common virulence factor of the accessory genome of African *S. aureus* (Lina et al., 1999; Lebughe et al., 2017).

### Genotyping

All isolates were *spa* typed; multilocus sequence typing (MLST) was done exemplarily for one isolate of each *spa* type. Related MLST sequence types (ST) were grouped in clonal clusters (CC) if they shared at least six of the seven alleles of the MLST housekeeping genes as implemented in eBURST (http://eburst.mlst.net/).

The concatenated sequences of the seven MLST genes were used to construct a Neighbor-Joining (NJ) tree as implemented in MEGA7 (www.megasoftware.net). The phylogenetic distance between groups was calculated using the Maximum Composite Likelihood method. Additional STs were included in the phylogenetic tree. They comprise major STs of *S. aureus* in Africa (ST15, ST121, ST152) (Schaumburg et al., 2014a), an early branching *S. aureus* from the DR Congo (ST2353) (Schaumburg et al., 2015), *S. aureus* from Gabonese bats (ST2984, ST3259, ST3301, ST3302) (Held et al., 2017), *S. argenteus* (ST75, ST850, ST1304, ST1850, ST2198) (Ng et al., 2009; Schuster et al., 2017), and *S. schweitzeri* (e.g., ST1700, ST1822, ST2296, ST2465) (Schaumburg et al., 2012a, 2015).

To analyze the position of the isolates in the overall *S. aureus* population, we screened the *S. aureus* MLST Database website (http://pubmlst.org/saureus/, sited at the University of Oxford) for the most closely related STs. These STs and others from our study were used to construct a minimum spanning tree (MST) as implemented in the SeqSphere^+^ software (version 2.4.0, Ridom GmbH, Münster, Germany).

## Results

In total, 250 fecal samples were collected from six roosting sites (Figure 1). Due to the sampling method, we were unable to assign one fecal sample to individual bats. To rule out multiple isolates from one bat (sampling bias), we included one isolate per *spa* type per sampling site and date in the final analysis. Overall, 53 isolates were included. Resistance to penicillin and intermediate susceptibility to tetracycline were detected in one isolate, each. The remaining isolates (*n* = 51) were susceptible to oxacillin, levofloxacin, glycopeptides, daptomycin, fosfomycin, linezolid, erythromycin, clindamycin, gentamycin, rifampicin, and trimethoprim/sulfamethoxazole (Table 1).

**Table 1:** Molecular characteristics of *Staphylococcus aureus* complex from bats, Nigeria, 2015–2016.

**Clonal complex (*n*)**	**Sequence type (*n*)**	***spa* types (*n*)**	**Species**	**PVL (*n*)**	**Antimicrobial resistance (*n*)**	**Sampling site**
CC1725 (27)	ST1725 (1)	t16686 (1)	*S. aureus*	Negative (1)	None	Student Union Building
	ST1726 (10)	**t16693** (1), **t16697** (3), **t16701** (1), t16703 (1), **t16704** (2), **t16733** (1), **t16734** (1)	*S. aureus*	Positive (8)	None	Student Union Building, Amphi-Theatre
	ST3958 (3)	NT (1), **t16681** (1), **t16696** (1)	*S. aureus*	Positive (2)	Tetracycline (1)	Student Union Building
	ST3959 (5)	**t16700** (3), **t16687** (1), t16702 (1)	*S. aureus*	Positive (4)	None	Student Union Building, Library
	ST4013 (1)	**t16695** (1)	*S. aureus*	Positive (1)	Penicillin (1)	Student Union Building
	ST4043 (2)	**t16685** (1), t16756 (1)	*S. aureus*	Positive (1)	None	Student Union Building, Amphi-Theatre
	ST4047 (5)	**t15966** (5)	*S. aureus*	Positive (5)	None	Student Union Building, Library, Health Center
CC2463 (9)	ST2463 (1)	NT (1)	*S. schweitzeri*	Negative (1)	None	Student Union Building
	ST3962 (4)	t16680 (1), t16682 (1), t16688 (1), t16694 (1)	*S. schweitzeri*	Negative (4)	None	Student Union Building, Amphi-Theatre, Library
	ST4316 (4)	t16684 (4)	*S. schweitzeri*	Negative (4)	None	Student Union Building, Library, Health Center
CC3960 (2)	ST3952 (1)	t17074 (1)	*S. argenteus*	Negative (1)	None	Student Union Building
	ST3960 (1)	t17079 (1)	*S. argenteus*	Negative (1)	None	Health Center
CC3961 (8)	ST3961 (4)	t16748 (3), t16755 (1)	*S. argenteus*	Negative (4)	None	Student Union Building, Library, Health Center
	ST3963 (1)	NT (1)	*S. argenteus*	Negative (1)	None	Student Union Building
	ST3980 (3)	t16747 (3)	*S. argenteus*	Negative (3)	None	Student Union Building, Library, Health Center
Singletons (7)	ST2465 (1)	t16732 (1)	*S. schweitzeri*	Negative (1)	None	Student Union Building
	ST2467 (1)	t5725 (1)	*S. schweitzeri*	Negative (1)	None	Library
	ST3964 (1)	**t16683** (1)	*S. aureus*	Positive (1)	None	Student Union Building
	ST4326 (4)	t16757 (4)	*S. argenteus*	Negative (4)	None	Student Union Building, Amphi-Theatre, Library, Health Center

*NT, non-typeable; spa types of PVL-positive isolates in bold*.

**Figure 2:**
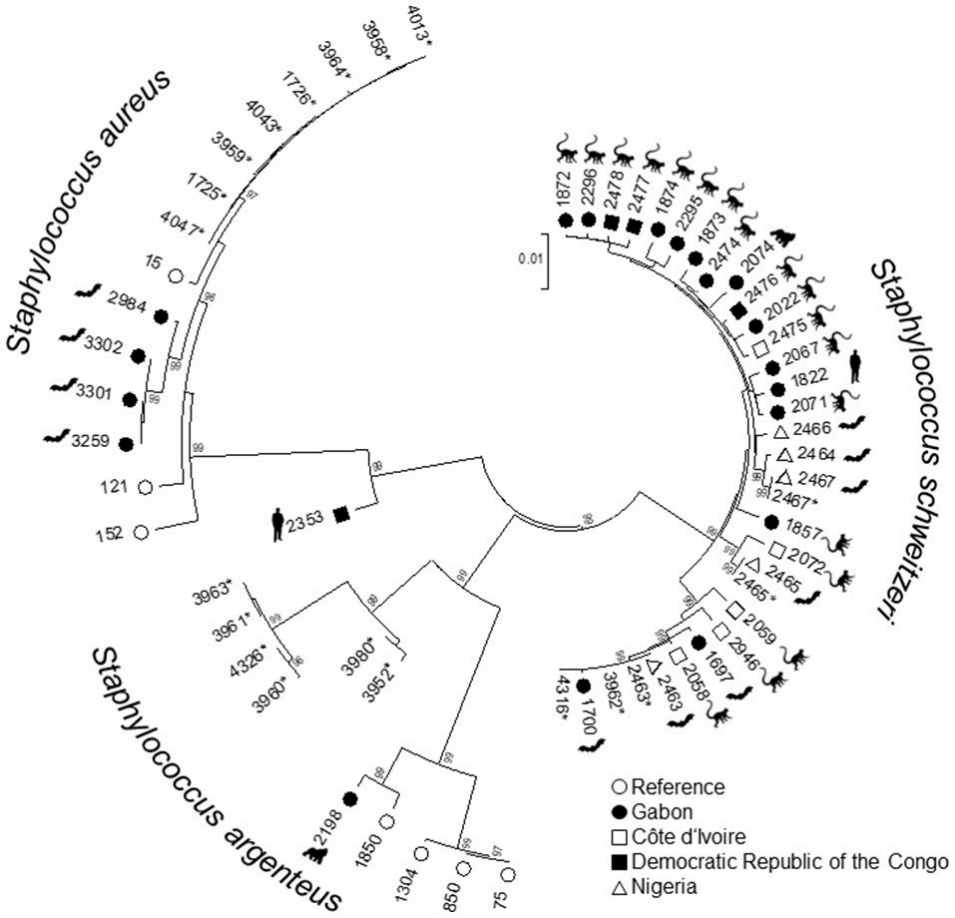
Genetic relatedness of *Staphylococcus aureus* complex from bats in Nigeria. A Neighbor-Joining-Tree was constructed using the concatenated sequences of the multilocus sequence typing (MLST) scheme. Isolates from this study (^*^) were combined with sequence types (ST) associated with *S. aureus, Staphylococcus schweitzeri*, and *Staphylococcus argenteus*. Labels show the respective STs. The hosts of the reference isolates (e.g., bats, monkeys, gorillas, humans) are indicated. Only bootstrap values of ≥95 (inferred from 500 replicates) are shown next to the branches.

All isolates were identified as *S. aureus* by MALDI-TOF. However, the isolates were reclassified as *S. aureus* (*n* = 28, 52.8%), *S. argenteus* (*n* = 14, 26.4%), and *S. schweitzeri* (*n* = 11, 20.8%) based on the presence of *nuc* and the genealogical clustering of the concatenated MLST alleles (Figure 2). All isolates were negative for *scn, sak*, and *chp* genes.

In total, 31 *spa* types (plus three non-typable isolates) and 19 STs were detected (Table 1). The three non-typable isolates belonged to *S. aureus, S. schweitzeri*, and *S. argenteus*. The predominant STs in *S. aureus* (*n* = 28) was ST1726 (35.7%, *n* = 10), followed by ST4047 (17.9%, *n* = 5) and ST3959 (17.9%, *n* = 5). STs associated with *S. schweitzeri* (*n* = 11) were ST3962 (36.4%, *n* = 4), ST4316 (36.4%, *n* = 4) ST2463, ST2465, and ST2467 (one isolate each). *S. argenteus* (*n* = 14) was associated with ST3961 (28.6%, *n* = 4), ST4326 (28.6%, *n* = 4), ST3980 (21.4%, *n* = 3), ST3952, ST3960, ST3963 (one isolate each). Apart from ST2463, ST2465, and ST2467, all other STs have not been reported (Table 1; Akobi et al., 2012). The three major CCs were CC1725 (*S. aureus*), CC2463 (*S. schweitzeri*), and CC3961 (*S. argenteus*, Table 1). PVL-positive isolates (*n* = 22) were only detected among *S. aureus*, of which 78.6% were PVL positive and associated with CC1725.

The concatenated sequences of the seven housekeeping genes included in the MLST scheme were used to construct a NJ tree (Figure 2). To assess the phylogenetic position of the isolates from this study within the *S. aureus* complex, we included additional STs associated with *S. schweitzeri, S. argenteus*, and African *S. aureus* (see materials and methods). All three species were separated into different clades supported by high bootstrap values (Figure 2). The mean distances between the *S. aureus* isolates and *S. argenteus* and *S. schweitzeri* was 0.1 and 0.08 base substitutions per site, respectively. STs of *S. schweitzeri* from this study were closely related with isolates from bats (ST2464) in Nigeria and Gabon (ST1700) or monkey from Côte d'Ivoire (ST2072, Figure 2). In contrast, both *S. aureus* and *S. argenteus* from this study were grouped in distinct clades that were separated from the clades of the reference STs (Figure 2).

**Figure 3:**
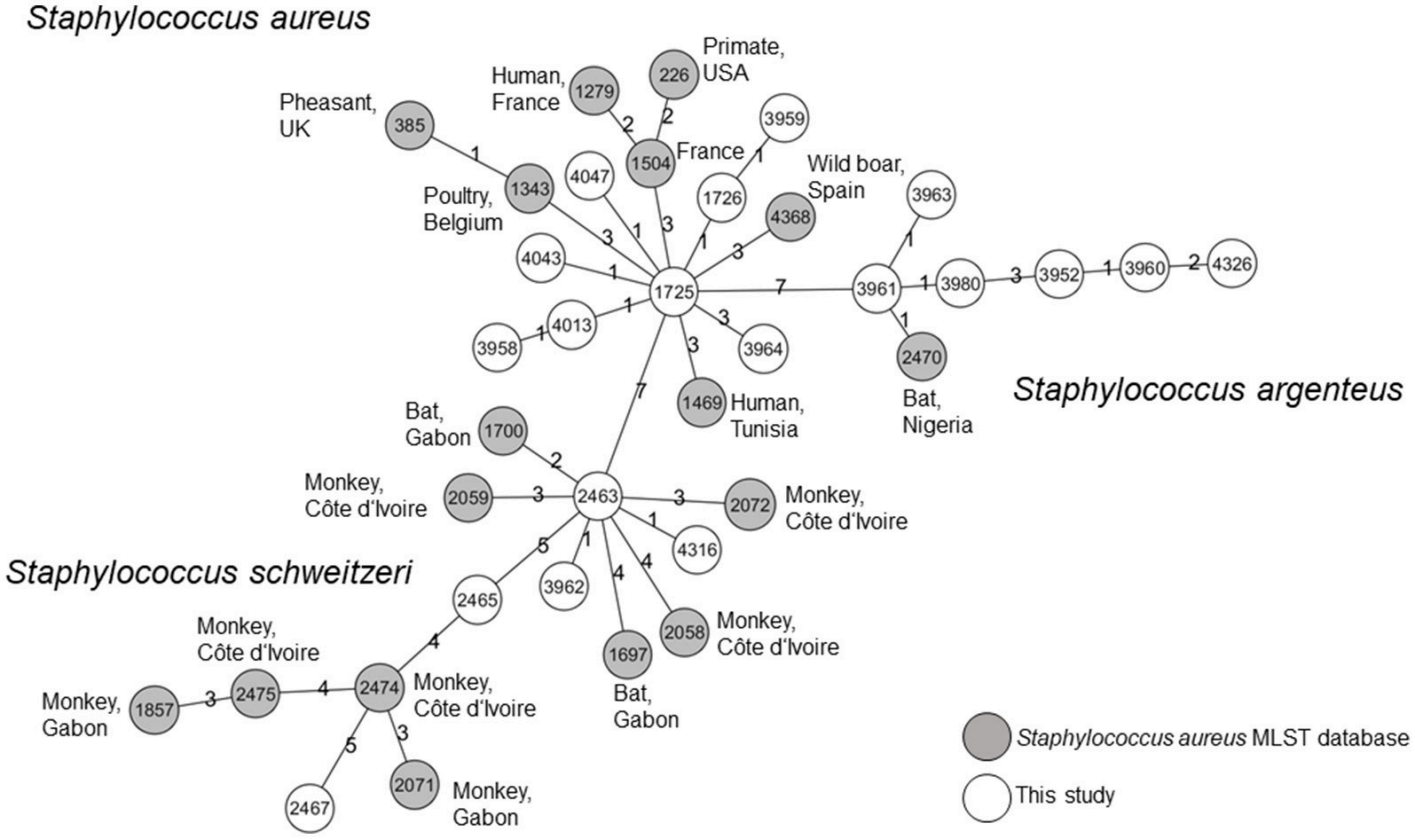
Minimum spanning tree. All isolates form this study and the most closely related STs as published on the *Staphylococcus aureus* MLST Database website (https://pubmlst.org/saureus/) were used to construct this tree based on the seven housekeeping genes of the MLST scheme. Nodes are labeled with STs. The origin of the isolates is color-coded (white: this study, gray: MLST database). The source (host and country) are indicated for all STs from the MLST database. The distance between the nodes is shown as the number of differing alleles.

In addition, we browsed the *S. aureus* MLST Database to identify isolates with the most closely related allele patterns to isolates from our study. The allelic profiles of these isolates and the isolates of our study were used to constructed a MST. *S. aureus, S. schweitzeri*, and *S. argenteus* were separated in three clusters. These clusters did not share any of the MLST alleles (Figure 3). *S. aureus* from this study shared three alleles with its closest relative. *S. schweitzeri* and *S. argenteus* shared two and one alleles, respectively with the closest related STs.

## Discussion

We analyzed the population structure of *S. aureus* complex from fecal samples of fruit bats (*E. helvum*) in Ile-Ife, Nigeria. The main findings are the presence of *S. argenteus* and a cross-species transmission and wide geographical spread of *S. schweitzeri* among African wildlife. Apart from two *S. aureus*, all isolates were susceptible to the antibiotics investigated here. This is in line with recent studies which showed that antimicrobial resistance is almost absent in African wildlife (Akobi et al., 2012; Held et al., 2017), but can be detected if animals had close contact with humans (i.e., in sanctuaries; Schaumburg et al., 2012b). Human contact with *E. helvum* in OAU is restricted though systematic culling activities take place due to the destruction of trees by these migratory mammals. The limited exposure of the isolates to human hosts is further supported by the absence of *scn, sak*, and *chp* genes. The staphylococcal complement inhibitor (*scn*), staphylokinase (*sak*), or chemotaxis inhibitory protein (*chp*) can specifically modulate the innate immune system of humans, and are considered as mechanisms of *S. aureus* to adapt to the human host (van Wamel et al., 2006; Senghore et al., 2016).

It is noteworthy that the proportion of PVL-positive *S. aureus* was high (78.6%) in our study. Previous investigations revealed a low prevalence of PVL (0–3.5%) in *S. aureus* from African wildlife (e.g., monkeys, bats; Akobi et al., 2012; Schaumburg et al., 2012a). In general, due to the high prevalence of PVL-positive *S. aureus* (17–74%) among isolates from humans (Okon et al., 2007; Breurec et al., 2011; Schaumburg et al., 2011; Egyir et al., 2014), sub-Saharan Africa is now considered a “PVL-endemic region” (Schaumburg et al., 2014a). On the one hand, the high PVL-rate in our study might mirror a process of equilibration between rates in humans and bats. On the other hand, our finding might reflect a selection bias as we only included one isolate per *spa* type per sampling site per day. PVL-positive *S. schweitzeri* have not been reported yet. This is, however, surprising, since *S. schweitzeri* is mainly distributed in regions, where a high proportion of *S. aureus* is PVL-positive. A transfer of PVL-carrying phages to *S. schweitzeri* could therefore be possible. An acquisition of PVL has been shown, at least for *S. argenteus*: older isolates were PVL-negative, but there are now increasing reports of PVL-positive *S. argenteus* (Holt et al., 2011; Dupieux et al., 2015; Chantratita et al., 2016).

All three species were grouped into corresponding clades in the NJ tree (Figure 2). The *S. schweitzeri* isolates from this study were closely related to isolates from monkeys and bats from Côte d'Ivoire, Gabon, and Nigeria suggesting cross species and geographical spread of *S. schweitzeri*. Since monkeys and bats can share similar habitat, a cross-species transmission appears to be feasible. This intense geographical dispersal of similar clones seems to be a characteristic trait of *S. sch*weitzeri (Held et al., 2017). In contrast, *S. aureus* and *S. argenteus* from Nigerian bats were phylogenetically distinct from the reference isolates. This might point toward a clonal expansion of certain clones among bats and limited (if any) transmission between humans. Indeed, no ST of this study has been detected in humans particularly in clinical infection (Okon et al., 2007; Shittu et al., 2011, 2012). However, the separation of bat-related ST from reference strains might also be due to the low number and low diversity of the reference isolates included in the analysis. Future studies are therefore needed for a more detailed picture of the population structure of *S. aureus* and particularly *S. argenteus* in African wildlife. The minimum spanning tree further highlights that the majority of isolates from this study are unrelated to isolates published so far in the *S. aureus* MLST database.

The microbiome of fruit bats is complex and depends on the body habitat with higher diversities in urine compared to feces or saliva (Dietrich et al., 2017). The predominant phyla in the fecal microbiome of bats are Proteobacteria, Firmicutes, and Actinobacteria (Dietrich et al., 2017). Among the gram-positive bacteria. *Enterococcus* sp. *Lactococcus* sp. and *Staphylococcus* sp. are commonly isolated from feces/guano. They can become aerosolized possibly facilitating a transmission to other hosts (Borda et al., 2014). Interestingly, also rare *Staphylococcus* species (i.e., *Staphylococcus nepalensis*) are found in guano (Vandžurová et al., 2013).

Our study has some limitations: First, we were unable to associate isolates with individual bats and are therefore not able to quantify the colonization rate. Since we applied rigorous inclusion criteria (based on *spa* typing) to rule out a sampling bias, we might therefore rather underestimate the overall burden of *S. aureus* complex in bats. Second, we did not apply whole genome sequencing due to limited funding opportunities for microbiological research in Africa. Comparing whole genome data of isolates from bats and humans would be valuable to identify host associated genetic elements, which might play a role in host adaptation (Lowder et al., 2009; Murray et al., 2017; Strauß et al., 2017). In conclusion, we found a high proportion of *S. schweitzeri* isolates from bats in Nigeria. The absence of antimicrobial resistance and immune evasion cluster suggest a limited exposure of these isolates to the human host.

## Author contributions

AS and FS: designed the study; AO: performed sampling and culture based microbiological analyses; AM, FO, and FS: did the molecular analyses and sequence based genotyping; AS, KB, AM, and FS: analyzed the data; AO, AS, and FS: drafted the manuscript. All authors reviewed and agreed on the final version of the manuscript. All authors have commented and agreed on the final version of the manuscript.

### Conflict of interest statement

The authors declare that the research was conducted in the absence of any commercial or financial relationships that could be construed as a potential conflict of interest.
